# Tongue Pressure and Grip Strength as Indicators of Persistent Dysphagia After Acute Stroke

**DOI:** 10.1007/s00455-024-10766-3

**Published:** 2024-10-28

**Authors:** Miho Ohashi, Yoichiro Aoyagi, Tatsuya Iwasawa, Kumiko Sakaguchi, Tomonari Saito, Yuki Sakamoto, Daisuke Ishiyama, Kazumi Kimura

**Affiliations:** 1 Department of Rehabilitation Medicine, Graduate School of Medicine, Nippon Medical School, 1-1-5 Sendagi, Bunkyo-ku, Tokyo, 113-8603, Japan; 2 Department of Rehabilitation Medicine, Nippon Medical School Hospital, Tokyo, Japan; 3 Department of Neurology, Nippon Medical School, Tokyo, Japan

**Keywords:** Acute stroke, Dysphagia, Sarcopenia, Tongue pressure, Grip strength, Skeletal muscle mass

## Abstract

This study aimed to identify the independent predictors of postacute stroke dysphagia at discharge using sarcopenia-related parameters. This single-center prospective observational study assessed consecutive inpatients diagnosed with cerebral infarction or cerebral hemorrhage upon admission to the stroke unit. Tongue pressure, grip strength, and body composition were evaluated within 48 h. Dysphagia was defined by a functional oral intake scale of ≤ 5. Patient characteristics were compared between non-dysphagia and dysphagia groups using Mann–Whitney or chi-squared tests. Logistic regression analysis was performed using age, sex, tongue pressure, grip strength, skeletal muscle mass index (SMI), and National Institutes of Health Stroke Scale (NIHSS) scores as explanatory variables, with dysphagia at discharge as the objective variable. A total of 302 patients (mean age: 69.4 ± 13.8 years, 67.5% male) were analyzed, with 64 having dysphagia at discharge (21.2%). The dysphagia group was significantly older (*p*<0.001), had higher NIHSS scores on admission (*p*<0.001), lower SMI (*p* = 0.002), lower grip strength (*p*<0.001), and lower tongue pressure (*p*<0.001) than the non-dysphagia group. Logistic regression revealed that age (OR: 1.042, *p* = 0.018), tongue pressure (OR: 0.954, *p* = 0.010), and grip strength (OR: 0.943, *p* = 0.048) on admission were independent predictors of dysphagia at discharge, while NIHSS scores (OR: 1.403, *p* = 0.106), sex, and SMI (OR: 1.403, *p* = 0.150) were not. Older age, reduced tongue pressure, and reduced grip strength are strong predictors of persistent poststroke dysphagia at discharge. Thus, muscle strength is a more valuable parameter than muscle mass in predicting persistent poststroke dysphagia.

## Introduction

The age of stroke onset has become higher in the past years [1]. From the 1970s, median age has dropped from 50s to 70s (70 and 77 years for men and women, respectively) in Japan [2, 3]. Sarcopenia and frailty, which are common in older people, have been reportedly connected with activities of daily living events after stroke [4, 5]. However, how sarcopenia or frailty is related to poststroke dysphagia remains unclear.

Dysphagia is a common adverse event occurring after acute stroke. Prevalence of its poststroke type at onset is 37–78% [6–10], 27% at 7 days [11], 20% at 3–4 weeks [12], and 2–18% at 6 months after stroke onset [11, 13]. Persistent dysphagia after stroke affects subsequent treatment decisions. The Guideline for the Early Management of Acute Ischemic Stroke states that for this category of patients, nasogastric tubes for feeding are required in the early phase of stroke (first 7 days). In contrast, percutaneous gastrostomy tubes should be placed in patients with longer periods of dysphagia (> 2–3 weeks) [9]. Accordingly, patients with poststroke dysphagia are reported to experience prolonged rehabilitation requiring further hospitalization postdischarge [14, 15]. In Japan, the average duration of acute conditions is 16 days [16]. Therefore, predicting the persistence of poststroke dysphagia is an important focus of the research for reducing the need for the placement of percutaneous gastrostomy tubes or further rehabilitation postdischarge. However, only a few reports predicted persistent dysphagia 2–3 weeks after stroke onset or at hospital discharge. The National Institute of Health Stroke Scale (NIHSS), bilateral lesions, dysarthria [14], and skeletal muscle mass [17] have been described as possible factors having an impact on persistent dysphagia; however, these are retrospective cohort studies. Thus, data available on this issue are limited.

We hypothesized that parameters evaluated to diagnose sarcopenia may be associated with poststroke dysphagia. Thus, in our study, skeletal muscle mass and grip strength, which are included in the sarcopenia diagnostic criteria [18, 19] and tongue pressure [20] representing muscle strength in swallowing were measured prospectively in patients with acute stroke at admission. This study aimed to determine independent predictors of poststroke dysphagia at discharge.

## Methods

### Study Design and Patient Enrollment

This study was conducted using a single-center prospective observational design. Consecutive inpatients who had cerebral infarction or cerebral hemorrhage during admission to the stroke unit at Nippon Medical School Hospital between June 2022 and August 2023 were analyzed. Exclusion criteria were as follows: (a) death during hospitalization, (b) isolation due to infectious diseases such as coronavirus disease 2019 and tuberculosis, (c) inability to follow instructions for measuring grip strength and/or tongue pressure due to consciousness problems (Japan coma scale > II-10) and/or higher cerebral dysfunction, and (d) missing data in medical history. A written informed consent was obtained from patients or legal representatives for the further registration of medical records. The study was approved by the ethics committee of Nippon Medical School Hospital and has been performed in accordance with the ethical standards laid down in the 1964 Declaration of Helsinki and its later amendments.

### Patient Characteristics

Data on age, sex, and body mass index (BMI) on admission and medical history of stroke were included in a registry database and prospectively analyzed. Moreover, stroke severity was evaluated based on the NIHSS score on admission. The stroke type (ischemic or hemorrhagic), modified Rankin Scale (mRS) score before admission, and duration of hospitalization were also considered the assessed variables. Patients with ischemic stroke were categorized as per the Trial of Org 10172 in Acute Stroke Treatment (TOAST) criteria [21].

### Physical Function and Body Composition Evaluation

Grip strength and body composition were assessed within 48 h of admission. The grip strength was measured twice for each hand using a Smedley hand dynamometer (TAKEI, Niigata, Japan), and the maximum value was used for evaluation [17]. The body composition analysis of a skeletal muscle mass involved the use of a bioelectrical impedance analysis device (InBody S10; InBody, Tokyo, Japan) in a supine position, and skeletal muscle mass index (SMI) was obtained as a ratio of the determined skeletal muscle mass and the squared height in meters.

### Swallowing Assessment

Repetitive saliva swallowing test (RSST) [22, 23], modified water swallowing test (MWST) [24, 25], and tongue pressure tests were conducted within 48 h of admission.

RSST is a screening test, in which a patient is instructed to swallow saliva for the maximum number of times for 30 s, while deglutition is assessed through palpation of the larynx. Two or fewer dry swallows detected within 30 s are regarded as abnormal. MWST was developed to identify aspiration after swallowing 3 mL of water. Three mL of cold water was administered to an oral vestibule of a patient who was instructed to swallow it. If a patient was unable to swallow or had dyspnea, coughing, or wet-hoarse dysphonia, an appropriate score was assigned (1 for inability to swallow, 2 for dyspnea, and 3 for cough or dysphonia), and the test was finished. Otherwise, a participant was instructed to perform 2 dry swallows. If the water could be swallowed but neither of the 2 dry swallows could be carried out within 30 s, a score of 4 was given. For the ability to perform the water and 2 dry swallows, a score of 5 was obtained. Thus, a scale of 1–5 was involved in the MWST evaluation, where the lowest score represented the most severe dysphagia. A score of less than 4 was regarded as abnormal [24, 25].

Tongue pressure was evaluated using a tongue pressure measuring device (TPM-02E, JMS Co., Ltd., Hiroshima, Japan) [26, 27] along with a disposable oral balloon probe device. Using this device, elevating air pressure between the front part of a palate and a tongue can be measured when a balloon at the tip of a plastic pipe probe is compressed at a midpoint of a patient’s central incisors with closed lips for 5 s with maximum effort. The obtained values for pressure were recorded 3 times with 30-s intervals and averaged [26, 27]. The swallowing assessment was performed by speech–language–hearing therapists.

### Study Outcome

Primary outcome was the presence or absence of dysphagia at discharge from the hospital. According to a previous study, dysphagia was defined as a functional oral intake scale (FOIS) level of ≤ 5 [26, 28, 29] that ranges from level 1 (nothing by mouth) to level 7 (a full unlimited oral diet). A FOIS level 5 indicates the ability to swallow in an oral diet with different consistencies but with the need for special preparation. The FOIS was analyzed at four-time points: before, on (within 48 h), 7 days post-admission, and at discharge. The evaluation was performed by speech–language–hearing therapists in accordance with a consumption or food and liquid consistency rate advised by objective swallowing evaluations.

### Statistical Analysis

Differences in clinical characteristics between the two groups of patients were initially assessed using Mann–Whitney and chi-squared tests for continuous and categorical variables, respectively.

Multiple logistic regression analyses were performed with dysphagia at discharge as the objective variable and tongue pressure, grip strength, and SMI as explanatory variables. Considering clinical assumptions regarding the potential significance, age, sex, and NIHSS score were also included in the explanatory variables. Data are presented as odds ratios (ORs) with 95% confidence intervals (CIs). The variance inflation factor (VIF) was estimated to calculate multicollinearity.

Spearman’s rank correlation coefficient was also assessed to establish the link between tongue pressure, grip strength, SMI, and age. All analyses were performed with the SPSS version 29 statistical software (IBM Corp., Armonk, NY, USA). *p* < 0.05 was regarded as statistically significant.

## Results

**Fig. 1:**
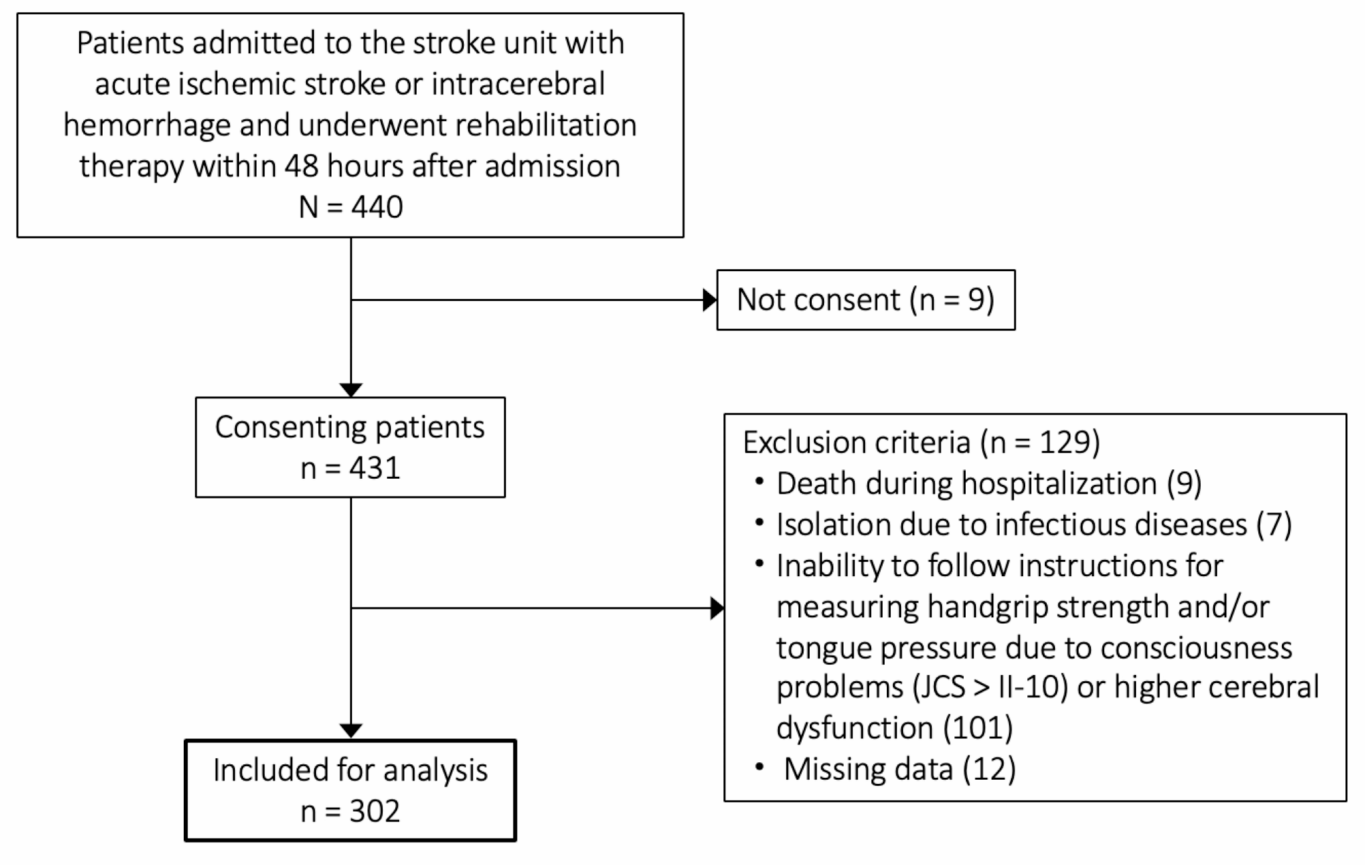
Flow chart of the patient enrollment process. *Abbreviation* JCS, Japan coma scale

We enrolled 440 consecutive patients with acute ischemic stroke or intracerebral hemorrhage who were admitted to the stroke unit at Nippon Medical School Hospital and underwent rehabilitation therapy within 48 h after admission (Fig. 1). Nine refused to provide consent, and 129 were excluded according to the outlined criteria. Consequently, 302 patients were included in the analysis. FOIS distributions of 302 patients on admission, at 7 days, and discharge are reflected in Table 1. Dysphagia defined as a FOIS level of ≤ 5 was found in 161 (53.3%), 110 (36.4%), and 64 (21.2%) patients on admission, at 7 days, and at discharge, respectively. None of the patients exhibited dysphagia on preadmission.

**Table 1:** Distribution of FOIS on preadmission, on admission, at 7 days, and at discharge

FOIS (level)		Preadmission (n)	Admission (n)	7 days (n)	Discharge (n)
1		0	23	10	11
2		0	5	2	0
3		0	0	1	0
4		0	0	1	1
5		0	133	96	52
6		49	34	46	57
7		253	107	146	181

*Abbreviation* FOIS, functional oral intake scale

In the univariate analysis, patients with dysphagia at discharge differed significantly in age, and preadmission mRS, and had higher NIHSS scores on admission than those from the non-dysphagia group (Table 2). Furthermore, patients with dysphagia had significantly lower SMI and grip strength. Considering the swallowing assessment, the RSST, MWST, and tongue pressure values were significantly lower in patients affected by dysphagia. Sex, prior stroke, stroke classification, BMI, and length of hospital stay did not differ significantly between the two groups.

**Table 2:** Comparison of characteristics between patients with dysphagia and non-dysphagia at discharge

	Total (N = 302)			Non-dysphagia (n = 238)			Dysphagia (n = 64)			*P*-value
Age (y.o.)	69.4	±	13.8	67.5	±	13.6	76.8	±	11.9	< 0.001
Sex (male)	204		(67.5)	166		(69.7)	38		(59.4)	0.133
Preadmission mRS	0.4	±	1.0	0.3	±	0.8	1.0	±	1.4	< 0.001
Prior stroke	54		(17.9)	40		(16.8)	14		(21.9)	0.362
Stroke classification										0.166
Large artery atherosclerosis	49		(16.2)	40		(16.8)	9		(14.1)	
Small-vessel occlusion/Lacune	51		(16.9)	44		(18.5)	7		(10.9)	
Cardioembolism	49		(16.2)	38		(16.0)	11		(17.2)	
Stroke of other determined etiology	44		(14.6)	36		(15.1)	8		(12.5)	
Stroke of other undetermined etiology	53		(17.6)	43		(18.1)	10		(15.6)	
Hemorrhage	56		(18.5)	37		(15.6)	19		(29.7)	
NIHSS on admission (total score)	3		(1–7)	2		(1–6)	5		(3–11)	< 0.001
BMI (kg/m^2^)	24.3	±	12.0	24.7	±	13.4	22.8	±	3.9	0.113
SMI (kg/m^2^)	6.8	±	1.3	6.9	±	1.2	6.3	±	1.4	0.002
Grip strength (kg)	23.3	±	9.8	24.6	±	9.4	18.2	±	9.5	< 0.001
Days to swallowing screening (day)	1.4	±	1.2	1.5	±	1.2	1.3	±	1.3	0.374
RSST (times)	3		(2–4)	3		(2–5)	2		(1–3)	< 0.001
MWST	5		(4–5)	5		(4–5)	4		(3–4)	< 0.001
Tongue pressure (kPa)	28.0	±	11.2	29.5	±	11.0	22.1	±	9.9	< 0.001
Preadmission FOIS	7		(7–7)	7		(7–7)	7		(6–7)	0.004
FOIS on admission	5		(5–7)	6		(5–7)	5		(5–5)	< 0.001
FOIS at 7 days	6		(5–7)	7		(6–7)	5		(5–5)	< 0.001
FOIS at discharge	7		(6–7)	7		(7–7)	5		(5–5)	< 0.001
Length of hospital stay (day)	17.6	±	9.6	17.1	±	9.0	19.2	±	11.3	0.166

Data are presented as n (%), mean ± standard deviation, or median (interquartile range) unless otherwise indicated

*Abbreviation* mRS, modified Rankin Scale; NIHSS, National Institutes of Health Stroke Scale; BMI, body mass index; SMI, skeletal muscle mass index; RSST, Repetitive Saliva Swallowing Test; MWST, modified water swallowing test; FOIS, functional oral intake scale

Multiple logistic regression analysis (Table 3) demonstrated that older age, lower tongue pressure, and lower grip strength on admission were independent predictors of dysphagia at discharge, whereas NIHSS, sex, and SMI were not. No significant multicollinearity (VIF = 1.068–3.016) was detected.

**Table 3:** Multivariable logistic regression analysis of dysphagia at discharge

	OR	(95% CI)		*P*-value
Age	1.042	(1.007–1.079)		0.018
Female	0.948	(0.365–2.461)		0.913
NIHSS	1.045	(0.991–1.103)		0.106
Tongue pressure (kPa)	0.954	(0.920–0.989)		0.010
Grip strength (kg)	0.943	(0.889–0.999)		0.048
SMI (kg/m^2^)	1.403	(0.885–2.225)		0.150

*Abbreviation* OR, odds ratio; CI, confidence interval; NIHSS, National Institutes of Health Stroke Scale; SMI, Skeletal Muscle Mass Index

**Fig. 2:**
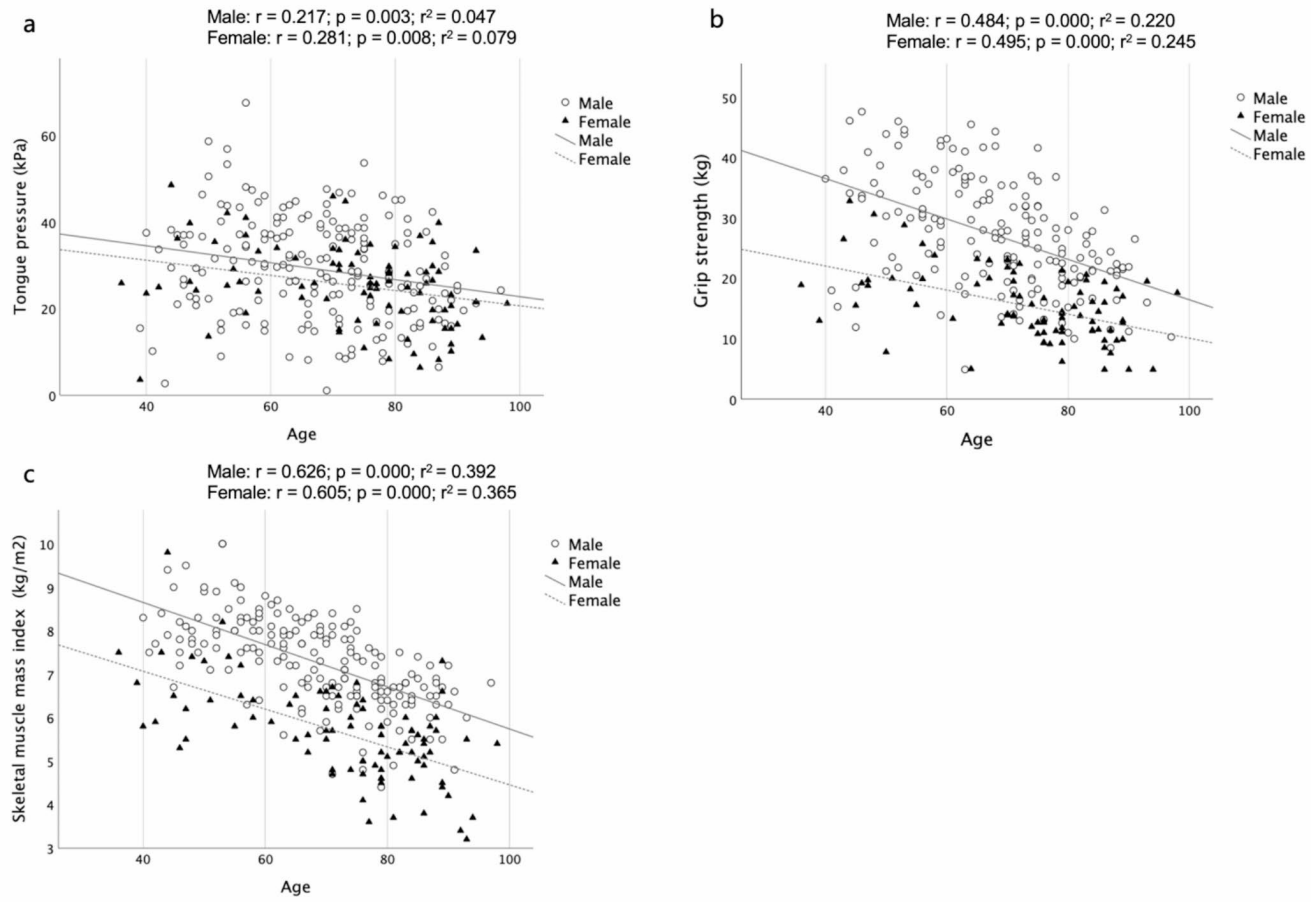
(*a*) Scatter plots of tongue pressure and age with correlation coefficients in male and female subgroups (*b*) Scatter plots of grip strength and age with correlation coefficients in male and female subgroups (*c*) Scatter plots of skeletal muscle mass index and age with correlation coefficients in male and female subgroups

Figure 2 illustrates scatter plots of (**a**) tongue pressure, (**b**) grip strength, (**c**) SMI, and age with correlation coefficients in male and female. SMI negatively correlated with and had the highest coefficient with age (*r* = 0.626 for male, *r* = 0.605 for female), whereas grip strength (*r* = 0.482 for male, *r* = 0.458 for female) and tongue pressure (*r* = 0.217 for male, *r* = 0.281 for female) showed moderate to low correlation.

## Discussion

This is the first study demonstrating that reduced tongue pressure and grip strength, independent of aging can accurately predict persistent poststroke dysphagia at discharge from the hospital. Notably, SMI and NIHSS on admission were not identified as independent predictors.

Reduced grip strength has been associated with an increased risk of all-cause death [30]. In the diagnostic algorithms elaborated by the European Working Group on Sarcopenia in Older People 2 [19] and the Asian Working Group for Sarcopenia 2019 [18], grip strength is the important parameter judging “no sarcopenia” and “sarcopenia probable.” Sarcopenia is described as an age-related, involuntary loss of skeletal muscle mass and strength [31, 32], while an aging-related decline in muscle strength appearing before the decrease of muscle mass refers to dynapenia [33]. The outcomes of this study propose a risk of persistent poststroke dysphagia not only in patients with sarcopenia as a background factor but also in those with dynapenia.

Tongue pressure generation promotes bolus propulsion during swallowing [34]. Low tongue pressure therefore causes oral and pharyngeal bolus residues after swallowing [35], which may lead to bolus aspiration. There are two possible reasons for low tongue pressure. Tongue pressure is decreased in persons with sarcopenia and dynapenia [36, 37]. This means that even a patient unaffected by dysphagia with a background of sarcopenia or dynapenia before stroke onset is likely to have reduced tongue pressure at preadmission. Hypoglossal nerve palsy accompanied by stroke is another cause of decreased tongue pressure. Sarcopenia/dynapenia accompanied by hypoglossal nerve palsy could provoke markedly reduced tongue pressure.

The result of SMI not being an independent predictor for persistent dysphagia is of interest. We suggest two possible reasons for this. First, age, which showed a high negative correlation with SMI (Fig. 2c), appears to be a more perceptive predictor than SMI in this logistic regression analysis. Second, low tongue pressure and grip strength, which are the characteristics of dynapenia and are likely to occur before SMI becomes low in association with sarcopenia, may have already caused persistent dysphagia.

This study has a few limitations. First, patients, in whom the measurements of tongue pressure or grip strength could not be performed due to higher cerebral dysfunction and/or impaired consciousness causing lack of cooperation, were excluded. These criteria were applied because a highly homogenous cohort was necessary to evaluate the relationship between tongue pressure/grip strength pressure and dysphagia. A study analyzing the predictors of dysphagia in patients with acute stroke with higher cerebral dysfunction and/or impaired consciousness is therefore needed in the future. Second, this study was conducted in a single center, and the results may not apply to the groups with varying demographic characteristics. However, we believe that there is no disparity in outcomes on the prevalence of poststroke dysphagia compared to previous studies, and the data do not appear to be markedly biased. Third, this study evaluated persistent dysphagia up to discharge from the acute hospital. Factors that predict further long-term persistence of dysphagia also need to be further assessed.

This study found that age, tongue pressure, and grip strength analyzed within 48 h of admission were independent predictors of persistent dysphagia at hospital discharge. Measuring tongue pressure and grip strength in addition to NIHSS at admission can help predict this pathologic condition and consider the treatment, such as placing percutaneous gastrostomy tubes, or further dysphagia rehabilitation after discharge.

## Data Availability

Raw data for this project are not publicly available in order to preserve participants’ privacy.
